# Serum Fibroblast Growth Factor 21 Level Is Associated with Aortic Stiffness in Patients on Maintenance Hemodialysis

**DOI:** 10.1155/2022/7098458

**Published:** 2022-02-11

**Authors:** Liang-Te Chiu, Chi-Di Hung, Lin Lin, Yu-Li Lin, Bang-Gee Hsu

**Affiliations:** ^1^Division of Nephrology, Hualien Tzu Chi Hospital, Buddhist Tzu Chi Medical Foundation, Hualien, Taiwan; ^2^School of Medicine, Tzu Chi University, Hualien, Taiwan; ^3^Division of Gastroenterology, Hualien Tzu Chi Hospital, Buddhist Tzu Chi Medical Foundation, Hualien, Taiwan

## Abstract

**Background:**

Fibroblast growth factor 21 (FGF-21) is a hormone that regulates glucose and lipid metabolism. High serum FGF-21 levels are associated with carotid atherosclerosis and coronary artery disease. This cross-sectional study aimed to assess the relationship between serum FGF-21 levels and carotid-femoral pulse wave velocity (cfPWV) in patients on maintenance hemodialysis (HD).

**Methods:**

Blood samples and baseline characteristics were collected from 130 HD patients. Serum FGF-21 concentrations were measured with an enzyme-linked immunosorbent assay kit. Aortic stiffness was defined as a carotid-femoral pulse wave velocity (cfPWV) of more than 10 m/s.

**Results:**

Of the 130 HD patients, aortic stiffness was diagnosed in 54 (41.5%). Serum FGF-21 levels were significantly higher in those with aortic stiffness than those without (*P* < 0.001). The FGF-21 level was independently associated with aortic stiffness (odds ratio (OR): 1.008; 95% CI: 1.003–1.012; *P*=0.001) after adjusting for diabetes mellitus, age, hypertension, C-reactive protein, and body weight in multivariable logistic regression analysis. Multivariable forward stepwise linear regression analysis also confirmed that the logarithmically transformed FGF-21 level (*β* = 3.245, 95% CI: 1.593–4.987, *P* < 0.001) was an independent predictor of cfPWV values. The area under the receiver operating characteristic (ROC) curve predicting aortic stiffness by the serum FGF-21 level was 0.693 (95% CI: 0.606–0.771, *P* < 0.001).

**Conclusions:**

Serum FGF-21 level positively correlates with cfPWV and is also an independent predictor of aortic stiffness in maintenance HD patients.

## 1. Introduction

Cardiovascular disease contributes to nearly half of deaths in patients with end-stage renal disease (ESRD) [1]. Patients with ESRD have a more than 10-fold increase in cardiovascular mortality, especially in younger individuals, compared with the general population [2]. Cardiovascular changes include accelerated atherosclerosis and arteriosclerosis with increased arterial stiffness [3]. The measurement of arterial stiffness helps assess cardiovascular risk and the development of therapeutical interventions. The carotid-femoral pulse wave velocity (cfPWV) is the established standard for measuring aortic stiffness, and it predicts cardiovascular and overall mortality in the ESRD population [4].

Fibroblast growth factor 21 (FGF-21) is a predominantly liver-derived hormone involved in regulating glucose and lipid metabolism, acting as a downstream target of peroxisome proliferator-activated receptor (PPAR) *α* and PPAR*γ*. It exerts versatile metabolic actions by binding to the FGF receptor and *β*-klotho, an extensively expressed transmembrane protein [5]. FGF-21 modulates gluconeogenesis, ketogenesis, insulin sensitivity, mitochondrial function, thermogenesis, and lipid metabolism [6, 7]. It has favorable metabolic effects in mice [8, 9]; in humans, however, higher circulating FGF-21 concentrations are associated with dyslipidemia and diabetes [10–12]. As a member of the FGF family, FGF-21 levels rise progressively during chronic kidney disease [11, 13]. Its association with inflammation, arterial stiffness, and increased cardiovascular burden has been reported [14, 15], suggesting that FGF-21 has potential as a marker for arterial stiffness in selected populations. Thus, the present study aimed to examine the association of FGF-21 and cfPWV in regular hemodialysis (HD) patients.

## 2. Materials and Methods

### 2.1. Patients

We recruited 130 HD patients from Hualien Tzu Chi Hospital Dialysis Center between March and July 2016. All subjects were over 20 years old and had undergone regular HD for at least 3 months. High-flux polysulfone disposable artificial kidneys (FX class dialyzer; Fresenius Medical Care, Bad Homburg, Germany) were used in the dialysis unit. Patients with malignancy, stroke, acute infection, amputated limbs, or who were bed-ridden were excluded from the study. Blood pressure was taken before HD sessions. A diagnosis of diabetes mellitus (DM) or hypertension was made if a patient was documented or taking insulin, hypoglycemic, or antihypertensive medications. Medical records were reviewed for the etiology of ESRD, prescription of angiotensin II receptor blockers, calcium channel blockers, *β*-blockers, statins, or fibrates. The research was carried out in accordance with the World Medical Association Declaration of Helsinki. The study protocol was approved by the Research Ethics Committee, Hualien Tzu Chi Hospital, Buddhist Tzu Chi Medical Foundation (IRB103-136-B), and informed consent was obtained from each subject.

### 2.2. Anthropometric Analysis and Biochemical Determinations

Height and post-HD body weight were measured, and body mass index (BMI) was calculated [16, 17]. According to the criteria specified by the Department of Health in Taiwan, overweight was defined as BMI ≥24 and obese as BMI ≥27 [18]. Fasting blood samples were collected and centrifuged at 3000 RPM for 10 min before HD. Serum samples were stored at 4°C and analyzed within 1 h of collection. Serum levels of blood urea nitrogen, creatinine, total cholesterol, triglyceride, glucose, total calcium, phosphorus, and C-reactive protein (CRP) were measured by standard laboratory methods (Siemens Advia 1800, Siemens Healthcare GmbH, Henkestr, Germany) [16, 17]. The fractional clearance index for urea (Kt/V) was calculated from a formal, single-compartment dialysis urea kinetic model. Serum FGF-21 (Phoenix Pharmaceuticals, Inc., Burlingame, CA, USA) concentrations were measured with commercially available enzyme immunoassay kits [19]. Serum intact parathyroid hormone (iPTH) levels were measured by the enzyme-linked immunosorbent assay (Abcam, Cambridge, MA, USA) [16, 17].

### 2.3. Carotid-Femoral Pulse Wave Velocity Measurements

cfPWV values were obtained by transcutaneous recording of the pressure pulse waveform in the target artery using applanation tonometry (SphygmoCor system, AtCor Medical, NSW, Australia). These measurements were performed with the patient in the supine position after a 10-min rest in a quiet, temperature-controlled room with simultaneous electrocardiographic (ECG) recordings. Pulse wave recordings were obtained consecutively at two superficial artery sites (carotid-femoral segment). Each set of pulse waves was processed with the Integral software (SphygmoCor system, AtCor Medical, NSW, Australia). ECG data were used to calculate the mean time difference between successive R-waves and pulse waves for each beat over an average of 10 consecutive cardiac cycles. The carotid-femoral distance was determined by subtracting the distance from the carotid location to the suprasternal notch, from the distance between the suprasternal notch and the femoral site. The cfPWV was calculated by dividing the distance by the mean time difference between the two recorded points. Quality indices were set to ensure data uniformity. Increased central arterial stiffness was defined as a cfPWV value > 10 m/s, according to the 2018 European Society of Cardiology guidelines, as a conservative cutoff for an increased cardiovascular risk [20].

### 2.4. Statistical Analysis

All statistical analyses were done with SPSS (version 19.0; SPSS Inc., Chicago, IL, USA). The Kolmogorov–Smirnov test was performed to determine the normal distribution. Normally distributed data are expressed as the mean ± standard deviation, and differences between groups were evaluated by the independent samples *t*-test. Non-normally-distributed data are expressed as medians and interquartile ranges. The differences between groups were evaluated by Mann–Whitney *U* test. Categorical data were tested by the chi-square test and are expressed as frequencies (%). Skewed distributions were transformed using base-10 logarithms (log) for analysis. The correlation between clinical variables and cfPWV values was evaluated using simple linear regression analysis, and variables that were significantly correlated with cfPWV values were tested for independence using a multivariable forward stepwise regression analysis. Variables significantly associated with aortic stiffness were tested for independence by multivariate logistic regression analysis. A receiver operating characteristic (ROC) curve was used to calculate the area under the curve (AUC) to identify the FGF-21 level that predicts aortic stiffness in HD patients. *P* < 0.05 was considered statistically significant.

## 3. Results

### 3.1. Baseline Characteristics

**Figure 1:**
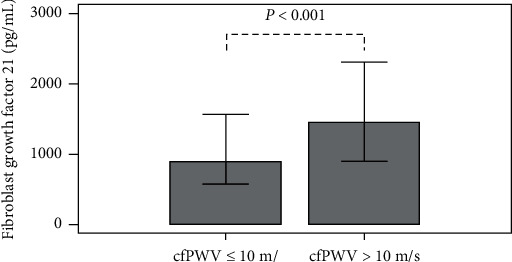
Comparison of fibroblast growth factor 21 levels between 130 hemodialysis patients with or without aortic stiffness.

The demographic and biochemical characteristics of the participants are summarized in Table 1. A total of 130 subjects, including 76 patients with a normal cfPWV (53.9% females, mean age = 61.8 ± 13.7 years) and 54 subjects with an increased cfPWV (42.6% females, mean age = 66.7 ± 12.0 years), were included. The etiologies of ESRD were diabetic nephropathy in 49 patients (37.7%), glomerulonephritis in 54 patients (41.5%), hypertensive nephropathy in 6 patients (4.6%), and other causes in 21 patients (16.2%). There were no differences in gender, HD duration, BMI in the obese or overweight range, history of smoking, Kt/V, diastolic blood pressure, and the use of antihypertensive or antihyperlipidemic therapy between the two groups. The blood urea nitrogen, creatinine, total cholesterol, triglycerides, fasting glucose, total calcium, phosphorus, and iPTH levels were similar between the two groups. Compared with subjects who had a normal cfPWV, subjects with an increased cfPWV were significantly older (*P*=0.036), had a higher body weight (*P*=0.027), BMI (*P*=0.048), systolic blood pressure (*P*=0.044), pulse pressure (*P*=0.001), CRP (*P*=0.040), FGF-21 (*P* < 0.001) (Figure 1), and a higher rate of DM (*P* < 0.001) and hypertension (*P*=0.026). Subjects with underlying diabetic nephropathy were more likely to have increased PWV (*P* < 0.001), while subjects with underlying glomerulonephritis were more likely to have normal PWV (*P*=0.030).

### 3.2. Serum FGF-21 Was Independently Associated with an Increased cfPWV (>10 m/s)

**Figure 2:**
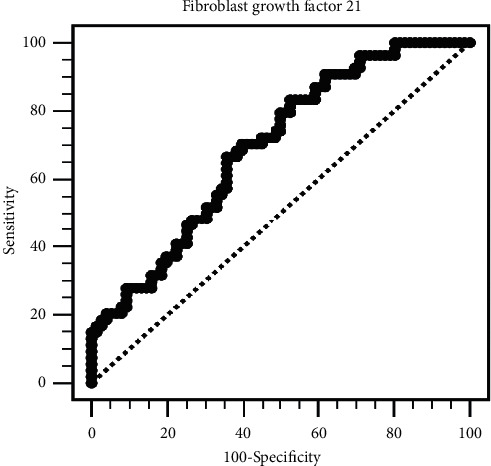
The area under the receiver operating characteristic curve indicates the predictive power of fibroblast growth factor 21 for aortic stiffness.

To determine the independent predictors for an increased cfPWV, we performed multivariable logistic regression analysis to determine the associations of FGF-21 and aortic stiffness (Table 2). FGF-21 was independently associated with an increased cfPWV (per 10 pg/mL, odds ratio (OR): 1.008; 95% confidence interval (CI): 1.003–1.012; *P*=0.001) after adjusting for other factors associated with increased cfPWV identified from Table 1 (DM, hypertension, age, CRP, and body weight). DM was also a predictor of increased cfPWV (OR: 4.269; 95% CI: 1.768–10.309; *P*=0.001). ROC analysis was performed to identify the cutoff values of FGF-21 for aortic stiffness, and the AUC was 0.693 (*P* < 0.001) (Figure 2), and at an FGF-21 cutoff level of 1063.5 pg/mL, the sensitivity was 70% and the specificity was 40%.

### 3.3. Correlations between cfPWV Levels and Clinical Variables

We analyzed the correlations between the cfPWV values and clinical parameters (Table 3). The cfPWV value was positively correlated with age (*r* = 0.194, *P*=0.027), systolic blood pressure (*r* = 0.186, *P*=0.034), pulse pressure (*r* = 0.272, *P*=0.002), logarithmically transformed CRP (log-CRP, *r* = 0.242, *P*=0.006), log-FGF-21 (*r* = 0.335, *P* < 0.001), and the presence of DM (*r* = 0.378, *P* < 0.001). Furthermore, the stepwise multivariable regression analysis showed the log-FGF-21 level to be an independent predictor for cfPWV (*β* = 3.245; 95% CI: 1.593–4.987, *P* < 0.001, Table 3), among others including DM status (*β* = 1.974; 95% CI: 0.918–3.031, *P*=0.001) and log-CRP levels (*β* = 0.978; 95% CI: 0.030–1.927, *P*=0.037). All other tested variables were not significant and were excluded.

## 4. Discussion

The most important finding of this study was the association of FGF-21 levels and cfPWV values, shown for the first time in regular HD patients.

Patients suffering from ESRD have increased arterial stiffness and cardiovascular mortality [21–23], resulting from traditional and nontraditional uremia-related cardiovascular risk factors. cfPWV has been reported as a strong independent predictor of overall and cardiovascular mortality in a hemodialysis population [4]. In general, arteries become stiffer with age and may develop atherosclerosis. The pathogenesis involves structural changes in the medial layer of the elastic arteries, such as the aorta and other major arteries. There is endothelial and smooth muscle dysfunction caused by inflammation and increased oxidative stress [21]. The most prevalent vascular findings are luminal narrowing, wall thickening, and reduced elasticity, that is, arteriosclerosis [24].

Factors related to diseases such as DM, hypertension, and chronic kidney disease and lifestyle habits such as smoking contribute to arterial stiffness beyond normal aging [21]. Advanced chronic kidney disease is associated with worse arterial stiffness than early disease [25]. Accelerated progression of arterial stiffness has been noted in the dialysis population; uremic factors, in addition to traditional risk factors, have been identified as independent determinants of this progression [26]. In line with the abovementioned reports, this study showed that aortic stiffness was correlated with DM, increased systolic blood pressure, pulse pressure, CRP, and age in regular HD patients.

Blood pressure is one of the most significant contributing factors to cfPWV [27]. In this study, cfPWV is correlated with systolic blood pressure and pulse pressure but not diastolic blood pressure or mean arterial pressure, showing that pulsatile blood pressure had more impact on cfPWV than the steady component. Similarly, a previous invasive study showed that pulse pressure had the best correlation with cfPWV among a group of normotensive and hypertensive subjects [28]. This increased pulsatility transmits to low resistance vascular beds, causing increased vascular mechanical strain and subsequent organ damage [29]. In our study, the association between cfPWV and age, systolic blood pressure, and pulse pressure disappeared after multivariate linear regression analysis, suggesting that DM, inflammation, and FGF-21 are more important determinants of arterial stiffness in the ESRD population with accelerated and deranged vascular aging.

Studies have shown that exogenous FGF-21 analog administration can significantly improve cardiometabolic profiles in obese or overweight diabetic patients [30–32]. This protective effect on the cardiovascular system is consistent with the beneficial effects of FGF-21 observed in animal studies. Nevertheless, the elevation of serum FGF-21 is associated with several vascular complications, including increased intima-media thickness, atherosclerotic plaque formation, and arterial stiffness [10, 15, 33, 34]. Whether the elevation of FGF-21 is beneficial or harmful to the cardiovascular system remains unclear. This paradoxical elevation could be a compensatory response to the underlying cardiovascular stress or FGF-21 resistance caused by the downregulation of target organ receptors [32, 35, 36]. Therefore, FGF-21 might serve as a potential marker for cardiovascular stress.

Studies showed that FGF-21 exerts protective effects against the development of atherosclerosis through the modulation of interactions between the adipose tissue, liver, and blood vessels [37], reducing vascular inflammation and oxidative stress [38]. In apolipoprotein E–/– mice, FGF-21 deficiency results in accelerated atherosclerosis and premature death, along with hypoadiponectinemia and hypercholesterolemia. Exogenous treatment with recombinant mouse FGF-21 induces adipocyte secretion of adiponectin, significantly reducing neointima formation, the proliferation of smooth muscle cells, and macrophage inflammation [37]. Hypercholesterolemia was also diminished via downregulation of the transcription factor sterol regulatory element-binding protein-2 in hepatocytes [37]. FGF-21 inhibits NLRP3 inflammasome-mediated vascular endothelial cells pyroptosis, possibly by improving mitochondrial function, reducing ROS production, and endoplasmic reticulum stress in the endothelial cells [39, 40]. An in vitro study revealed that FGF-21 protects macrophages against ox-LDL-induced foam cell formation and apoptosis by suppressing the CHOP expression [41]. Overall, FGF-21 exerts beneficial vascular effects, possibly by mitigating vascular inflammation and dyslipidemia, independent of its antiobese and antidiabetic activity. Hence, elevated serum FGF-21 associated with atherosclerosis likely reflects this adaptive mechanism [37, 38]. In patients with ESRD, serum FGF-21 levels may be up to 20 times normal levels [11]. FGF-21 is not dialyzable [42], suggesting that renal factors influence its serum level in ESRD patients. Higher levels of FGF-21 were associated with increased arterial stiffness as measured by flow-mediated dilatation in a study of patients undergoing continuous ambulatory peritoneal dialysis [14]. Moreover, higher FGF-21 levels were associated with a higher all-cause mortality rate but not cardiovascular events, in a Japanese study of HD patients [43].

Our study results should be interpreted considering several limitations. First, we cannot assume any causal associations due to the cross-sectional study design. Second, the sample size was small, and larger longitudinal studies will be needed for confirmation. Third, we only investigated the relationship between cfPWV and FGF-21. Other factors of note, such as intima-media thickness or left ventricular mass index, may provide additional invaluable information. Fourth, we did not measure residual renal function, which may influence the development of atherosclerosis. Fifth, a dietary survey may be needed since FGF-21 is maximally elevated under low protein and high carbohydrate intakes [44]. Sixth, there was no healthy control group for comparison.

## 5. Conclusion

Higher serum FGF-21 levels were associated with higher cfPWV values in a group of regular HD patients. This study suggests that serum FGF-21 levels may be a predictive marker of aortic stiffness in HD patients. Defining this new parameter might help stratify the cardiovascular disease risk and offer potential therapeutic strategies for atherosclerosis. Future studies such as interventional trials are warranted to determine the clinical significance of FGF-21.

## Figures and Tables

**Table 1:** Clinical characteristics of the study population.

Parameters	Overall (*n* = 130)	cfPWV ≤10 m/s (*n* = 76)	cfPWV >10 m/s (*n* = 54)	*P* value
Clinical characteristics				
Age (years)	63.8 ± 13.2	61.8 ± 13.7	66.7 ± 12.0	0.036^*∗*^
Hemodialysis duration (months)	59.6 (23.9–130.7)	80.0 (22.2–144.8)	56.5 (26.7–92.9)	0.232
Female, *n* (%)	64 (49.2)	41 (53.9)	23 (42.6)	0.202
Smoking, *n* (%)	18 (13.8)	8 (10.5)	10 (18.5)	0.303
Body weight (kg)	63.2 ± 14.6	60.8 ± 14.1	66.5 ± 14.7	0.027^*∗*^
Body mass index (kg/m^2^)	24.7 ± 4.8	24.0 ± 4.7	25.7 ± 4.9	0.048^*∗*^
Overweight or obese	67 (51.5)	37 (48.7)	30 (55.6)	0.480
Kt/V (gotch)	1.3 ± 0.2	1.4 ± 0.2	1.3 ± 0.2	0.200
Hemodynamic parameters				
Carotid-femoral PWV (m/s)	10.1 ± 3.3	7.8 ± 1.2	13.2 ± 2.6	<0.001^*∗*^
Systolic blood pressure (mmHg)	142.0 ± 25.9	138.2 ± 26.7	147.4 ± 23.8	0.044^*∗*^
Diastolic blood pressure (mmHg)	77.0 ± 15.7	77.5 ± 15.6	76.2 ± 16.0	0.658
Mean arterial pressure (mmHg)	98.6 ± 17.8	97.7 ± 18.2	100.0 ± 17.2	0.478
Pulse pressure (mmHg)	65.0 ± 18.0	60.7 ± 17.4	71.2 ± 17.0	0.001^*∗*^
Laboratory parameters				
Total cholesterol (mg/dL)	146.8 ± 34.6	148.3 ± 39.0	144.5 ± 27.6	0.535
Triglyceride (mg/dL)	114.0 (84.0–187.0)	108.5 (83.3–186.5)	122.0 (87.0–187.5)	0.406
Fasting glucose (mg/dL)	130.5 (109.8–167.3)	128.0 (106.3–151.0)	133.5 (110.8–182.0)	0.135
Blood urea nitrogen (mg/dL)	60.83 ± 15.00	61.72 ± 14.48	59.57 ± 15.76	0.423
Creatinine (mg/dL)	9.31 ± 2.07	9.42 ± 2.00	9.14 ± 2.18	0.456
Total calcium (mg/dL)	9.0 ± 0.8	8.9 ± 0.7	9.2 ± 0.8	0.067
Phosphorus (mg/dL)	4.8 ± 1.3	4.7 ± 1.3	4.8 ± 1.3	0.708
iPTH (pg/mL)	204.1 (69.4–462.7)	256.45 (106.4–475.2)	150.7 (51.2–462.7)	0.163
C-reactive protein (mg/dL)	0.26 (0.08–0.66)	0.23 (0.06–0.46)	0.38 (0.12–0.95)	0.040^*∗*^
FGF-21 (pg/mL)	1150.5 (728.7–1863.1)	887.5 (572.3–1579.8)	1433.1 (889.3–2317.7)	<0.001^*∗*^
Underlying disease				
Diabetes mellitus, *n* (%)	49 (37.7)	18 (23.7)	31 (57.4)	<0.001^*∗*^
Hypertension, *n* (%)	62 (47.7)	30 (39.5)	32 (59.3)	0.026^*∗*^
Etiology of ESRD				
Diabetic nephropathy, *n* (%)	49 (37.7)	18 (23.7)	31 (57.4)	<0.001^*∗*^
Hypertensive nephropathy, *n* (%)	6 (4.6)	4 (5.3)	2 (3.7)	1.000
Glomerulonephritis, *n* (%)	54 (41.5)	38 (50.0)	16 (29.6)	0.030^*∗*^
Other, *n* (%)	21 (16.2)	16 (21.1)	5 (9.3)	0.092
Medication				
ACE-inhibitors/ARBs, *n* (%)	38 (29.2)	19 (25.0)	19 (35.2)	0.208
*β*-blocker, *n* (%)	36 (27.7)	19 (25.0)	17 (31.5)	0.416
Calcium channel blocker, *n* (%)	50 (38.5)	30 (39.5)	20 (37.0)	0.778
Statin, *n* (%)	20 (15.4)	9 (11.8)	11 (20.4)	0.184
Fibrate, *n* (%)	15 (11.5)	8 (10.5)	7 (13.0)	0.668

Values for continuous variables are shown as mean ± standard deviation after analysis by student's *t*-test; variables not normally distributed are shown as median and interquartile range after analysis by Mann-Whitney *U* test; values are presented as number (%)after analysis by the chi-square test. Kt/V, fractional clearance index for urea; cfPWV, carotid-femoral pulse wave velocity; iPTH, intact parathyroid hormone; FGF-21, fibroblast growth factor 21; ESRD, end-stage renal disease; ACE, angiotensin-converting enzyme; ARB, angiotensin receptor blockers. ^*∗*^*P* < 0.05 was considered statistically significant.

**Table 2:** Multivariable logistic regression analysis of the factors correlated to aortic stiffness.

Variables	Odds ratio	95% confidence interval	*P* value
FGF-21, per 10 pg/mL	1.008	1.003–1.012	0.001^*∗*^
Diabetes mellitus, present	4.269	1.768–10.309	0.001^*∗*^
Age, per 10 years	1.412	0.986–2.024	0.060
Hypertension, present	3.105	1.279–7.540	0.012
C-reactive protein, 1 mg/dL	2.103	0.994–4.446	0.052
Body weight, 1 kg	1.027	0.995–1.061	0.093

Analysis of data was performed using the multivariate logistic regression analysis (adopted factors: diabetes mellitus, hypertension, age, body weight, C-reactive protein, and FGF-21). FGF-21, fibroblast growth factor 21. ^*∗*^*P* < 0.05 was considered statistically significant.

**Table 3:** Correlations between central pulse wave velocity and clinical variables and multivariable stepwise linear regression analysis of cfPWV.

Variables	Carotid-femoral pulse wave velocity (m/s)				
	Simple linear regression		Multivariable linear regression		
	*r*	*P* value	*β*	95% confidence interval	*P* value
Female	−0.113	0.200	—	—	—
Diabetes mellitus	0.378	<0.001^*∗*^	1.974	0.918–3.031	0.001^*∗*^
Hypertension	0.128	0.145	—	—	—
Age (years)	0.194	0.027^*∗*^	—	—	—
Log-HD duration (months)	−0.060	0.494	—	—	—
Body weight (kg)	0.162	0.066	—	—	—
Body mass index (kg/m^2^)	0.133	0.131	—	—	—
Systolic blood pressure (mmHg)	0.186	0.034^*∗*^	—	—	—
Diastolic blood pressure (mmHg)	−0.004	0.966	—	—	—
Mean arterial pressure (mmHg)	0.088	0.320		—	—
Pulse pressure (mmHg)	0.272	0.002^*∗*^		—	—
Total cholesterol (mg/dL)	−0.039	0.663	—	—	—
Log-triglyceride (mg/dL)	0.087	0.328	—	—	—
Log-fasting plasma glucose (mg/dL)	0.110	0.213	—	—	—
Total calcium (mg/dL)	0.116	0.189	—	—	—
Phosphorus (mg/dL)	0.056	0.529	—	—	—
Log-iPTH (pg/mL)	−0.153	0.082	—	—	—
Log-CRP (mg/dL)	0.242	0.006^*∗*^	0.978	0.030–1.927	0.037^*∗*^
Log-FGF-21 (pg/mL)	0.335	<0.001^*∗*^	3.245	1.593–4.987	<0.001^*∗*^
Kt/V (gotch)	−0.070	0.435	—	—	—

The data of HD duration, triglyceride, fasting plasma glucose, iPTH, CRP, and FGF-21 levels showed skewed distribution and therefore were log-transformed before analysis. Analysis of data was performed using the simple linear regression analysis or multivariate stepwise linear regression analysis (adopted factors: diabetes mellitus, age, systolic blood pressure, pulse pressure, log-CRP, and log-FGF-21). HD, hemodialysis; iPTH, intact parathyroid hormone; CRP, C-reactive protein; FGF-21, fibroblast growth factor 21; Kt/V, fractional clearance index for urea; *β*, unstandardized regression coefficient. ^*∗*^*P* < 0.05 was considered statistically significant.

## Data Availability

The data used to support the findings of this study are available from the corresponding author on reasonable request.
